# A low-latency graph computer to identify metastable particles at the Large Hadron Collider for real-time analysis of potential dark matter signatures

**DOI:** 10.1038/s41598-024-60319-9

**Published:** 2024-05-03

**Authors:** Ashutosh Vijay Kotwal, Hunter Kemeny, Zijie Yang, Jiqing Fan

**Affiliations:** 1 Department of Physics, Duke University, Durham, NC 27708 USA; 2 Department of Electrical and Computer Engineering, Duke University, Durham, NC 27708 USA

**Keywords:** Unsupervised learning, Fast pattern recognition, Low latency, Disappearing track trigger, Large Hadron Collider, Experimental particle physics, Imaging techniques, Information technology, Electrical and electronic engineering, Computational science

## Abstract

Image recognition is a pervasive task in many information-processing environments. We present a solution to a difficult pattern recognition problem that lies at the heart of experimental particle physics. Future experiments with very high-intensity beams will produce a spray of thousands of particles in each beam-target or beam-beam collision. Recognizing the trajectories of these particles as they traverse layers of electronic sensors is a massive image recognition task that has never been accomplished in real time. We present a real-time processing solution that is implemented in a commercial field-programmable gate array using high-level synthesis. It is an unsupervised learning algorithm that uses techniques of graph computing. A prime application is the low-latency analysis of dark-matter signatures involving metastable charged particles that manifest as disappearing tracks.

## Introduction

One of the challenges of machine intelligence is its application in use cases of high throughput and low latency. Since data often populate a high-dimensional parameter space, the classification function contains a huge number of parameters which can limit the computational speed. The evaluation of elaborate functions such as deep neural networks in software on generic CPUs is often replaced by porting the code to run on specialized hardware such as GPUs, resulting in substantial increase in throughput. Another approach is to use configurable logic blocks, distributed memory and digital signal processors on a field-programmable gate array (FPGA) to implement a dedicated algorithm. The advent of high-level synthesis (HLS), wherein a high-level programming language such as C/C++ can be used to code the algorithm in a manner amenable to an FPGA implementation, has helped to reduce the development time by automating the conversion of C/C++ code to a digital circuit on an FPGA.

Broadly speaking, applications of machine intelligence can be classified as supervised/reinforcement or unsupervised learning. Two requirements for the former are (1) the availability of high-quality training data, as exemplified by deep-learning models, and (2) external intervention or feedback during the training or testing phase. Supervised learning models can be executed with a deterministic latency. On the other hand, unsupervised learning methods typically have a data-dependent latency, but with the advantage of not having requirements (1) and (2).

This paper is novel in three respects. First, we present an implementation of an unsupervised learning method^1,2^ that executes with a fixed latency. Second, instead of software, we demonstrate a digital circuit implementation that executes with much higher throughput and lower latency than is possible in software. Third, the method is based entirely on a graph-computing architecture; all computations are uniformly distributed across all graph nodes with a complete absence of any central processor. Its highlights also include modularity, parallelizability and amenability to pipelining.

For a demonstration we choose an extreme use case for machine intelligence; the reconstruction of particle trajectories at the Large Hadron Collider (LHC), as described in^3^. High-density bunches of protons collide at 25 ns intervals, and each bunch crossing is expected to produce up to 200 individual proton-proton collisions on average at the high-luminosity LHC (HL-LHC). With each collision generating about 70 charged particles that pass through the cylindrical layers of fast, pixelated silicon sensors, tens of thousands of space points are generated at a rate of 40 MHz at the intersections of the particle trajectories with these layers^3^. Within this massive point cloud, occasionally there are embedded sets of space points associated with a handful of high-momentum charged particles; these signatures lasting for a few nanoseconds indicate the occurrence of rare quantum-mechanical processes. Such processes have identified fundamental laws of physics that govern the behavior of the building blocks of matter, their interactions via quantum forces, and the mass-generating effect called the Higgs mechanism (see^4,5^ for reviews).

An exciting possibility for the LHC is that similar quantum-mechanical processes might describe the production and decay of heavy particles associated with dark matter. If such processes were active soon after the Big Bang and were responsible for creating the observed dark matter relic density in the Universe, the LHC beam energy and collision rate may be sufficient to reproduce these processes in the laboratory (see references of^3^). The key question is—can we identify and filter out these rare, ephemeral traces from the enormous point clouds which are refreshed at 40 MHz? To date, no computational scheme has been operated to cope with this use case at the ATLAS and CMS experiments.

A potential solution to this challenge was proposed in^3^ using unsupervised machine learning based on graph computing. This algorithm proceeds in two steps. In the first step, the entire point cloud is sliced into one-dimensional (azimuthal boundaries only) or two-dimensional (azimuthal and longitudinal boundaries) wedges such that each wedge contains $N = 2^n$ space points on each of the concentric, cylindrical sensor layers. The overlap between these subsets may be tuned to optimize the acceptance for a high-momentum particle, i.e. the trajectory of such a particle of interest must be completely contained within one of the wedges. The boundaries of the wedges vary from event to event depending on the distribution of the points on the layers. The implementation of this “slicing” algorithm will be the topic of a future paper.

In this paper we describe the FPGA implementation of the second step whose conceptual design was discussed in^3^. This graph-computing algorithm performs unsupervised pattern recognition on the set of 3D space points in a wedge, such that the radially-distributed set of selected points is consistent with the sensor signals (“hits”) deposited by a high-momentum particle traversing a uniform, axial magnetic field.

We demonstrate that the graph-computing algorithm can be implemented in an FPGA to achieve the 40 MHz throughput with a latency of 250 ns. Charged-particle progenitors of dark matter may be produced at the LHC (see references of^3^), with the only visible signature being a charged-particle trajectory (“track”) if the progenitor decays invisibly into dark matter after traversing the silicon sensors, i.e. disappearing tracks. Since each beam crossing produces many megabytes of sensor data, the total data rate at the beam crossing frequency of 40 MHz exceeds the readout and processing capability by two orders of magnitude. Fortunately, a very large fraction of collisions do not produce the rare processes of physics interest. The essential technology for filtering out the small fraction of interesting collisions is “trigger” electronics that performs fast pattern recognition of sought-after signatures hidden in the sensor data. Such triggers have been operational for electrons, muons, photons and collimated particle flows called jets. However, a trigger for disappearing tracks has never been operational at the LHC, potentially preventing new physics from being discovered. Our trigger design is a significant step towards creating a disappearing track trigger with the requisite throughput and latency to handle the full bandwidth of the LHC experiments.

A disappearing-track trigger has to satisfy two requirements. First, a track must be found as soon as it is produced and before it decays. To satisfy this requirement, the track trigger must process information from the silicon pixel detector at the closest possible distance from the beam axis, typically within a radius of $\approx 25$ cm. For this reason we focus on track-triggering with a 5-layer pixel detector that is being built for the ATLAS and CMS experiments at the LHC. If the track trigger is based on sensors at larger radii, a significant loss in efficiency is incurred for interesting ranges of particle lifetime, as shown in^3^.

The second requirement of a disappearing-track trigger is that no charged particle or energy be detected at larger distances from the beam collision point, that can be associated with the trigger track in the pixel detector. This veto can be provided by existing calorimeter/muon-based triggers for high-momentum electrons, muons, protons, pions and kaons using the spatial and momentum correlation between the trigger signals. Optionally, the veto can be incorporated in the software-based filter at a later stage in the trigger chain. Hence we prioritize the first requirement of a real-time track trigger using the small-radius pixel detector.

We emphasize that our design is not limited or specific to the pixel detector; it is configurable for any number of layers at any radii, and for any geometry of the pixels or strips. Silicon strip-based detectors will be deployed by the ATLAS and CMS experiments in the radial range of 30–100 cm. Our design can be configured for general-purpose track reconstruction above a momentum threshold in the strip detector; this application will be considered in future work.

Detailed comparisons of our method with other methods based on associative memory, the Hough transform, neural networks, or tracklet-finding in paired sensors have been presented in Refs.^3,6^. The primary advantage of our method compared to the associative memory^7^, Hough transform^8^ and neural network^9^ techniques is that our method is intended to operate in the first-level trigger with an input rate of 40 MHz, while the latter are being pursued for the next trigger level with a lower input rate. The tracklet approach can process input data at 40 MHz but requires a special sensor configuration with pairs of closely-spaced strip layers^10^; our approach is compatible with any sensor configuration. A review of tracking triggers at the HL-LHC can be found in Ref.^11^.

## Methods

The methodology of^3^ is summarized here. We consider $L=5$ silicon pixel-sensor layers and $N = 2^n = 16$ hits per layer in the wedge; *L* and *N* are adjustable within FPGA resource constraints. With $L \times N$ coordinates as input, this algorithm finds smooth trajectories of particles. Each hit in the $N \times L$ grid is treated as a node in a graph. Graph-computing logic is used to compute discretized second derivatives (“laplacians”) at each node of the middle three layers. A particle’s helical trajectory is represented by a path through the graph that contains one node in each layer. As shown in^3^, reconstructing the trajectory corresponds to minimizing the (absolute value of the) laplacian at all nodes on this path.

A key insight of the algorithm is that the global minimum of all $(L-2)N^3$ laplacians is not found by searching for the local minima at each node. On the contrary, the algorithm succeeds by iteratively vetoing poor trajectories, i.e. rejecting the combinations of three nodes (triplets) that correspond to a large laplacian value. We refer to this method of convergence as pruning. The fraction of triplets that are rejected at each iteration can be tuned; following^3^, this implementation uses the fraction of 50%. Faster convergence would be achieved if the rejection fraction were say 75%—whether the robustness demonstrated in^3^ would be maintained for a larger rejection fraction is a topic for future study.

The number of links at a node with its two adjacent layers is initially $N^2$, so the total number of links is initially $(L-1)N^2$. These links are iteratively pruned, until only links that comprise smooth paths through all layers remain. Each iteration comprises two logical operations, pruning and consensus.

At the end of the iterative procedure, multiple trajectories are found in a wedge, most of which are “ghosts” and result from combinatorial chance. Ghost tracks zig-zag and do not satisfy smoothness criteria. A quality control procedure selects the smoothest trajectory. We show that the smoothest trajectory is always that of the high-momentum particle of interest, should one exist in the wedge. In the rare circumstance that there are two or more particles of interest in the wedge, simple extensions of the algorithm allow for further selection on the basis of momentum, smoothness and the desired number of tracks; these quantities are computed in the quality-control procedure. Thus it is straightforward to configure the quality control procedure to output all trajectories that satisfy trigger criteria.

### Pruning

Pruning is described in^3^ as follows: “The sort engine sorts the $N \times N$ list of $\Box _{ijk,l}$ values in increasing magnitude. Each $\Box _{ijk,l}$ value is stored as part of a tuple containing the associated *j* and *k* values which identify the corresponding triplet of hits. The sorted list of tuples is used by the scan engine to create a ranked list of *j* and *k* values, where the rank is defined as the ordinal number of first appearance in the sorted $\Box _{ijk,l}$ list. Thus, a *j* or *k* value with a large rank is one that never makes a smooth trajectory, while a low rank corresponds to a smoother trajectory. In each sort cycle, the *j* and *k* values with large rank are dropped, which purges those links that are unlikely to form smooth trajectories.” Here $\Box _{ijk,l}$ represents the 1D or 2D laplacian value as defined in^3^ for the triplet of hits (*i*, *j*, *k*), where hit *i* in layer $l \in \{1,\ldots ,L-2\}$ is linked to hit *j* in the next radial layer $(l+1)$ and hit *k* in the previous radial layer $(l-1)$. We implement the tuple as a 24-bit integer in which the upper 16 bits store the laplacian and the lower byte stores *j* and *k* in 4 bits each.

Pruning is an iterative and distributed algorithm. The iteration count *t* runs from $t = n$ to $t = 1$ and $t \rightarrow t-1$ for each successive iteration. For a given iteration, at each node *i* in layer *l*, there are approximately $2^t \times 2^t$ possible local paths connecting the nodes in layers $l-1$, *l*, and $l+1$. Each local path at node (*i*, *l*) consists of two links; one link to an outer node $(j,l+1)$ and one link to an inner node $(k,l-1)$. We denote these links as $(i,l;j,l+1)$ and $(i,l;k,l-1)$ respectively. This local path has the discrete laplacian value $\Box _{ijk,l}$. As described in^3^, pruning reduces the number of viable paths at each node to $2^{t-1} \times 2^{t-1}$, such that there are $2^{t-1}$ surviving $(i,l; j,l+1)$ links and $2^{t-1}$ surviving $(i,l;k,l-1)$ links.

A highlight of this paper is the implementation of the sort and scan engines that is fast, modular, parallelizable and amenable to pipelining. We prove (see section “Sort and scan engines”) that the combination of the sort and scan engines is mathematically equivalent to: (a) for each (*i*, *l*), construct a matrix of tuples indexed by the *j* and *k* values, (b) construct a MinimumFinder that finds the tuple with the smallest laplacian value in each row of this matrix, (c) the array of these minima, indexed by row, is processed by a Minimum Set Selector (MSS) circuit, which splits the array into two halves; of the $2^t$ minima, the lower (upper) half contains the smallest (largest) $2^{t-1}$ laplacians. Only the lower half are propagated to the next iteration, achieving the intended 50% rejection factor during pruning.

The symmetry between *j* and *k* (i.e. linked nodes in the adjacent sensor layers at larger and smaller radii respectively) is maintained by running in parallel a second MinimumFinder circuit on a transposed matrix of tuples.

The MSS design can be easily modified to reject say 75% at each iteration, by saving only the smallest $2^{t-2}$ laplacians. The design is efficient because no time or FPGA resources are wasted in further sorting, which is irrelevant at a given iteration due to the iterative nature of the algorithm.

### Consensus

As described in^3^, the consensus protocol is another crucial insight contributing to the success of the algorithm. The consensus protocol enables the local decisions at each node to be propagated to their linked nodes in adjacent layers so that the algorithm ultimately converges to the globally smoothest path. The consensus protocol is invoked after each iteration of pruning. Information percolates over time from each layer to more and more distant layers and a global vision over all layers is eventually achieved. In concert, all heavy-duty computations in the pruning step are local and distributed across all the nodes to be executed in parallel with low latency and high throughput.

In the consensus protocol, each link $(a, l_1; b, l_2)$ is compared with its partner link $(b, l_2; a, l_1)$ as maintained by the two respective nodes $(a,l_1)$ and $(b,l_2)$. If either link has been pruned by its respective node, the partner link is also eliminated from its linked node. The consensus protocol ensures that all surviving links are bi-directional, i.e. both nodes agree on their mutual link. Hence, after each iteration of pruning and consensus, the number of surviving links at each node is somewhat smaller than $2^t \times 2^t$.

### Quality control

At the end of this iterative algorithm, any surviving global path of length $L-1$ provides a linked list of nodes that serves as a reconstructed track^3^. Multiple tracks may be found in a wedge, most of which are ghosts. There is no assurance yet of the track quality—the goal of pruning and consensus is to find the smoothest possible tracks without any *a-priori* threshold on the smoothness.

A subsequent quality control procedure has been described in^3^. For both the first and second (signed) derivatives, crookedness is defined as the largest difference between any pair of nodes along a track. For example, the second derivative of a zig-zag track changes sign and will likely have a large value of crookedness. These metrics are computed separately for each dimension of 2D tracks. If the same track has the smallest value of crookedness for each of the four metrics, it is labelled as the smoothest track and selected as the final output of the algorithm.

Useful byproducts of the quality control procedure are the selected track’s curvature (inverse transverse momentum) and polar direction, as well as the four metrics of track quality. Trigger decision criteria can subsequently be applied to these quantities. It is straightforward to add a simple circuit to compute the track’s azimuthal angle.

**Figure 1:**
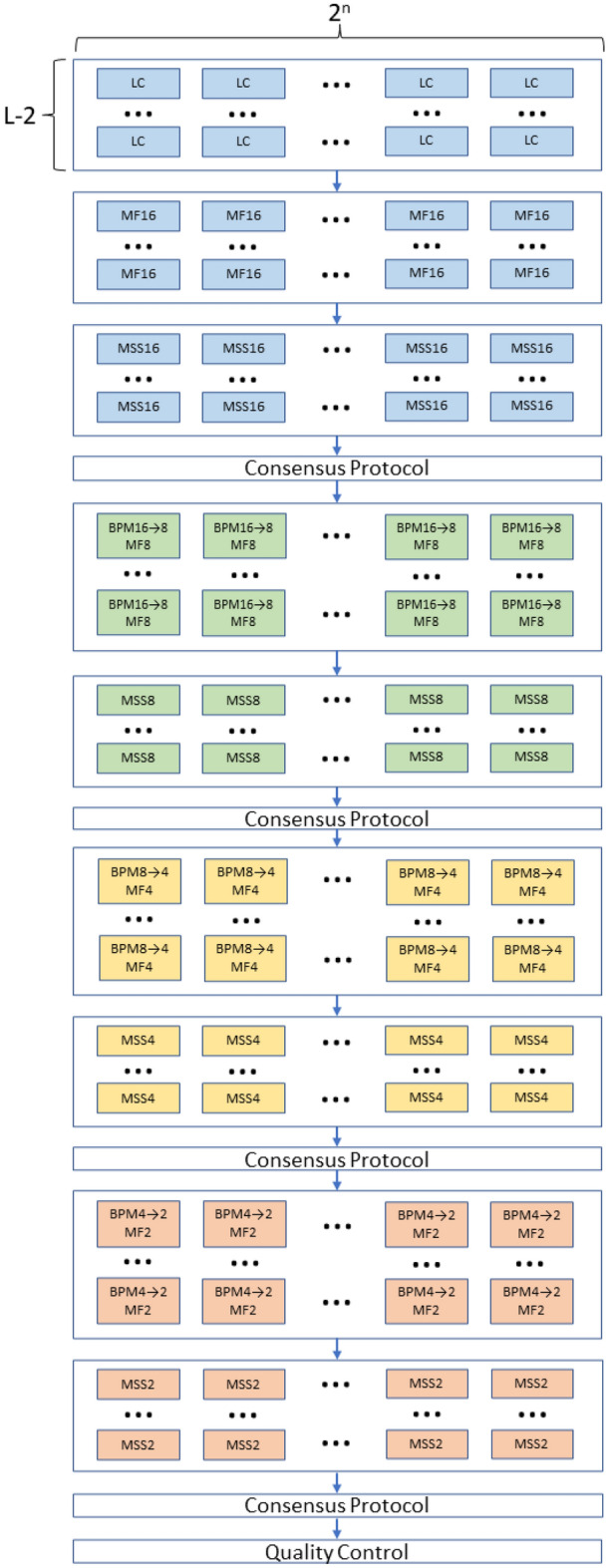
Block diagram of the data flow through the FPGA.

A possibility considered and resolved in^3^ is the intersection of two tracks. The solution involves an intervention after the second-last iteration to check for two smooth trajectories passing through a node. As the pruning executes at each node simultaneously, the required actions can be inserted into each node engine. Since the intersection of two smooth trajectories is a rare occurrence and can be resolved with a small addendum, the circuitry required for this intervention will be discussed in a future paper.

## Implementation

In this section we discuss the implementation details of the hardware modules. As shown in Fig. 1, the data flow through the following modules in sequence; laplacian calculator (LC), minimum finders (MF), maximum set selectors (MSS) and consensus protocol (CP). The latter three are chained *n* times for $t = n \ldots 1$. The final module is quality control (QC).

We implement the circuit using the xilinx vitis hls tool. vitis hls generates an RTL (register-transfer level) design of the digital network in Verilog and VHDL formats from its high-level C/C++ representation. These RTL formats can be used for programming an FPGA. Our results are presented using the xilinx FPGA XCVU19p-2-e, which has 4.1M lookup tables (LUT), 8.2M flip-flops (FF) and 3840 digital signal processors (DSP). All circuits are synchronous with an internal clock of 0.85 ns cycle time. Though a little faster than the recommended 1.1 ns clock cycle for this FPGA, it demonstrates the feasibility of a real-time track trigger.

In section “Resource usage” we show the hardware resource usage on the FPGA in terms of LUTs, FFs and DSPs, as well as module latencies according to the vitis synthesis.

### Laplacian calculator

The computation of $N^3(L-2)$ values of $\Box _{ijk,l}$ from $N \times L$ coordinates is shown in^3^ using weighted sums. The weighted coordinates incorporate the radial distances between layers, alignment corrections, and differences in resolution between the azimuthal and longitudinal dimensions. The weights also depend on whether the first or second derivative is being computed. For each hit there are three weighted coordinates for the three possible second derivatives (Eq. 7 of^6^), and two weighted coordinates for the forward and backward difference respectively (Eq. 6 of^6^). These five weighted coordinates for each hit position (per dimension) can be compacted into a long integer and stored in a lookup table.

Using the weighted coordinates as inputs, the LC uses only addition and the absolute value operation to compute the $N^3(L-2)$ tuples and save them in an $(L-2) \times N \times N \times N$ dimensional array TM. The tuple (laplacian, *j*, *k*) corresponding to one local path at node (*i*, *l*) is located at TM$[l-1][i][j][k]$.

The loops over *l* and *i* are unrolled so that the computations at each node proceed simultaneously in independent, replicated modules. In each module, the 3-term sum corresponding to the laplacian is split into two sequential pairwise sums. The latter are embedded inside a pipelined loop over *j* and an unrolled loop over *k*. A pairwise sum is performed by a DSP in one clock cycle.

The LC is designed for two-dimensional silicon sensors that measure both azimuthal ($\phi$) and longitudinal (*z*) coordinates. We represent these coordinates as 16-bit integers, which are passed to the LC as a bit-packed 32-bit word. In the LC, both coordinates are unpacked and their second derivatives are computed in a set of parallelized and pipelined DSPs. The final steps compute and add the respective absolute values, again using DSPs, to obtain the 2D laplacian $\Box _{ijk,l} = | \phi ^{\prime \prime }_{ijk,l} | + | z^{\prime \prime }_{ijk,l} |$ (for $l \in \{1,\ldots ,L-2\}$)^3^, and pack the 24-bit tuple. The difference in the sensors’ measurement resolution between the azimuthal and longitudinal coordinates has already been taken into account in their respective weighted values supplied to the LC. We expect 16-bit coordinates to provide adequate resolution of $\mathcal O$(1 $\mu$m) since wedge dimensions are expected to be smaller than 6 cm.

This design results in a 4-stage pipeline with $N = 2^n$ iterations over the pipeline, resulting in efficient (high duty factor) usage of LUTs, FFs and DSPs. With $N=16$ we achieve a latency of 21 ns (24 clock cycles) for the LC.

### Minimum finder

**Figure 2:**
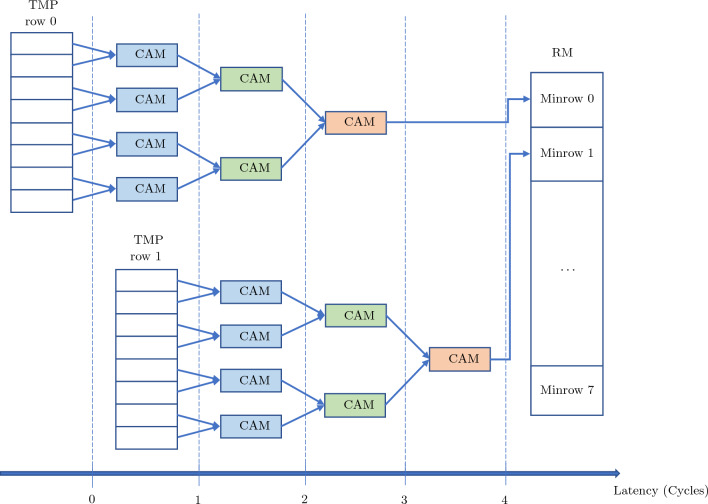
Block diagram of a pipelined MF8 corresponding to an MF built for $t=3$.

**Figure 3:**
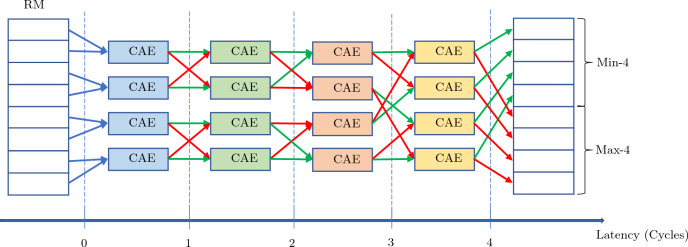
Block diagram of a pipelined MSS8 corresponding to an MSS built for $t=3$. The green (red) arrows represent the smaller (larger) of the two outputs of the respective CAE units.

The MF architecture is a pipeline of *t* stages, with each stage consisting of $2^{t-1}, 2^{t-2},\ldots , 1$ compare-and-minimize (CAM) units running in parallel. Each CAM outputs the smaller of its two input laplacians. The MF finds the minimum of $2^t$ inputs with a latency of 2*t* clock cycles. For each node (*i*, *l*) the row-wise minima of the 2D array TM$[l-1][i]$ are stored in a 1D array of length $2^t$ indexed by row. The loop over rows is pipelined to use a single MF and obtain an efficient architecture with a high duty factor. A second, identical MF processes the transpose of TM$[l-1][i]$ to obtain the column minima. The two MF circuits per node run concurrently. The 1D array of row minima (and equivalently, the column minima) is denoted as RM in Fig. 2 as the MF module’s output and in Fig. 3 as the MSS module’s input respectively.

The block diagram of a pipelined MF is shown in Fig. 2. Each MF uses $2^t -1$ CAM units. The latency of the MF is less than 29 clock cycles and reduces as both $2^t$ (due to pipelining) and as *t* (since the number of sequential internal stages $s = t$).

### Minimum set selector

The MSS is based on Batcher’s bitonic sorter^12,13^ that uses compare-and-exchange (CAE) units. Each CAE sorts its two inputs into ascending order. We implement an MSS that sorts $2^t$ inputs minimally so that the first $2^{t-1}$ values are the smallest.

Figure 3 shows a block diagram of a pipelined MSS. We take advantage of the pipelined design to process both the row-minima and the column-minima sequentially using a single MSS per node. It is possible to increase the duty factor by using the same MSS for multiple nodes, further increasing efficiency and reducing resource usage for a given latency requirement. The latency of the MSS is less than 29 clock cycles and reduces with *t* as $\approx t^2$, since the number of sequential internal stages $s = \frac{1}{2}(t-1)t + 1$. MSS uses $2^{t-1}s$ CAE units.

### Consensus protocol

The implementation of the CP is based on an array of booleans GL$[l-1][i][2][j]$ storing valid links between a node (*i*, *l*) and another node $(j,l \pm 1)$, where the sign is stored in the third (binary) dimension. GL contains a redundancy since for each pair of nodes in adjacent layers, the status of both unidirectional partner links, one directed radially outward and the other directed radially inward, are stored. This redundancy is an important aspect of the design since it enables a completely deterministic (data-independent) architecture and latency.

Consensus is imposed by setting both partner links to false if either of the partner links is false. This crucial step propagates locally-generated information in both directions along the tracks, enabling a globally-optimal decision.

### Build pruned matrix

As described above, all laplacians are computed once at the beginning of the wedge data flow into the circuit and stored in TM as one $2^n \times 2^n$ matrix per node. Starting with the second iteration of the algorithm, $t < n$, the MF process $2^t \times 2^t$ matrices of surviving paths and the MSS process $2^t$-length arrays. Thus we need to build pruned versions of TM for each node, TM$\rightarrow$TMP, with the lengths of the *j* and *k* dimensions each reduced by a factor of 2 (given our rejection factor of 50%). The $2^t \times 2^t$ TMP matrices per node serve as the inputs to MF for $t<n$.

Pruning eliminates $\frac{3}{4}$ of the local paths at each node. Therefore TM is initially a completely dense matrix and pruning and consensus increases its sparsity; with each iteration of pruning its density decreases by a factor of 4.

The purpose of the build-pruned-matrix (BPM) module is to compactify the sparsified version of TM to produce TMP which is smaller and almost completely dense. BPM uses the information on surviving links stored in the GL matrix to perform the compactification. BPM executes before MF in order to supply TMP to MF.

One of the goals of the algorithm proposed in^3^ is to make the FPGA circuit architecture, as well as its throughput and latency, completely independent of features of the data. All characteristics of the circuit should be *a-priori* deterministic and calculable. To this end, BPM defines TMP with fixed-length dimensions based on the deterministic nature of pruning.

The data dependency is handled by the consensus protocol. Another function of BPM is to propagate this information garnered by CP. One of the sub-modules of BPM sets the laplacians to $\infty$ in TMP for those local paths that are eliminated by CP. In this way, the data structures and logic circuits remain data-independent; the local paths flagged by CP for elimination are removed by the next iteration of pruning.

This factorization of functions is one of the insights presented in this paper as a way to handle all data with pre-determined circuits. One of the enabling features of this implementation is redundancy of critical information. In the case of BPM, the information in GL is partially replicated by storing the node indices of surviving links in redundant arrays. In practice, the additional memory usage is minimal and the benefit is substantial. The latency of BPM is less than 10 clock cycles and reduces with *t*.

### Quality control

The QC module consists of three sub-modules, findAllTracks (fAT), findBestTrack (fBT) and removeGhostTracks (rGT). We choose one of the *L* layers as the anchor layer at which tracks are defined; in practice, the layer that is radially in the middle is the most convenient. Iterating over all nodes in this layer, fAT creates a linked-list of nodes connected to each of these anchor nodes, thereby making a collection of tracks.

Next, fBT computes the four crookedness values along each of these tracks, as mentioned in section “Quality control”, using the node coordinates as inputs to DSPs to calculate first and second derivatives. Batcher’s bitonic sorters are used to find the smallest and the largest values of each metric; four sorters are deployed in parallel to ensure low latency. DSPs are used to calculate the crookedness values from these extrema.

Here again we encounter potential data-dependence in the number of track candidates. To eliminate data dependence, the fBT circuitry is replicated for each anchor node, regardless of whether a candidate track passes through that node. Typically, candidate tracks pass through half of the anchor nodes, implying that the other half of the fBT resources are wasted. The resource usage shown in Table 1 indicates that this cost is a small fraction of the total resources available. Hence we use this simple but effective solution to ensure a deterministic latency of the fBT sub-module. The array of booleans GL (see section “Consensus protocol”), which keeps a record of valid links between nodes, is used to flag and reject invalid track candidates subsequently.

**Table 1:** Timing performance and resource usage of various modules and sub-modules as estimated by synthesis using version 2020.2 of vitis hls.

Usage			Timing			Resources		
Block	Module	Pipelined function	Latency (cc)	Latency (ns)	Initiation interval (cc)	Digital signal processors	Flip-flops	Lookup tables
(full system)						4160	4,368,233	3,237,529
all_LC			24	20.60	24	3936	644,468	400,689
	LC $(\times 48)$		22	18.90	22	82	13,554	8320
		LC_$(D+D)$	2	1.70	1	2	768	130
		LC_$(16D+D)$	2	1.70	1	32	576	301
		LC_(16U$+$)	2	1.70	1	48	–	150
		LC_ET	1	0.85	1	–	–	7120
all_MF16			27	22.95	27	–	480,676	285,638
	MF16 $(\times 48)$		26	22.10	26	–	10,013	5653
		MF16_PS0 $(\times 2)$	1	0.85	1	–	392	320
		MF16_PS1 $(\times 2)$	1	0.85	1	–	196	160
		MF16_PS2 $(\times 2)$	1	0.85	1	–	98	80
		MF16_PS3 $(\times 2)$	1	0.85	1	–	49	40
all_MSS16			29	24.65	29	–	326,596	342,865
	MSS16 $(\times 48)$		27	22.95	27	–	6035	6833
		MSS16_PS $(\times 7)$	1	0.85	1	–	392	512
	CP		8	6.80	1	–	–	1024
all_MF8			29	24.65	29	–	546,101	627,548
	MF8 $(\times 48)$		27	22.95	27	–	5232	12,911
		BPM$16 \rightarrow 8$	10	8.50	10	–	1757	10,763
		MF8_PS0 $(\times 2)$	1	0.85	1	–	196	160
		MF8_PS1 $(\times 2)$	1	0.85	1	–	98	80
		MF8_PS2 $(\times 2)$	1	0.85	1	–	49	40
all_MSS8			19	16.15	19	–	112,372	196,993
	MSS8 $(\times 48)$		17	14.45	17	–	1956	3938
		MSS8_PS $(\times 4)$	1	0.85	1	–	196	256
	CP		4	3.40	1	–	–	1024
all_MF4			19	16.15	19	–	381,845	157,052
	MF4 $(\times 48)$		17	14.45	17	–	1810	3181
		BPM$8 \rightarrow 4$	6	5.10	6	–	463	2445
		MF4_PS0 $(\times 2)$	1	0.85	1	–	98	80
		MF4_PS1 $(\times 2)$	1	0.85	1	–	49	40
all_MSS4			13	11.18	13	–	41,284	89,809
	MSS4 $(\times 48)$		11	9.46	11	–	667	1777
		MSS4_PS $(\times 2)$	1	0.85	1	–	98	128
	CP		2	1.70	1	–	–	1024
all_MF2			12	10.20	12	–	321,509	83,132
	MF2 $(\times 48)$		10	8.50	10	–	553	1686
		BPM$4 \rightarrow 2$	4	3.40	4	–	137	1337
		MF2_PS0 $(\times 2)$	1	0.85	1	–	49	40
all_MSS2			9	7.65	9	–	15,574	90,450
	MSS2 $(\times 48)$		6	5.10	6	–	206	1538
		MSS2_PS $(\times 1)$	1	0.85	1	–	49	40
	CP		2	1.70	1	–	–	1024
QC	fAT		2	1.70	2	–	284,388	120,911
	fBT		24	20.62	24	224	98,936	71,689
	rGT		6	5.10	6	–	3860	86,302

In the “block” column, “all” refers to the collection over all $3 \times 16$ nodes in the graph, corresponding to 3 intermediate sensor layers and 16 hits per layer. This replication of the LC, MF and MSS modules is also indicated in the “module” column. In the “pipelined function” column, “PS*p*” refers to the $p^{\textrm{th}}$ pipeline stage of the minimum finders, and the replication of the pipeline stages in the MSS is indicated. The pipelined functions used in LC are described in section “Resource usage”. Initiation interval refers to the wait time until the circuit can process new data. Time delay in terms of the number of clock cycles is denoted by “cc”, where 1 cc $=$ 0.85 ns. The first row shows the total resources used by the entire system.

For each of the four crookedness metrics, fBT deploys a MF to find the track with the smallest crookedness value. If the same track is selected by all four criteria, fBT returns this track and its parameters as the output of the circuit.

The final sub-module rGT removes the remaining (ghost) tracks from the array GL by purging their associated links.

### Track parameters and metrics

As shown in^3^, the inverse of the particle’s momentum transverse to the beam axis (i.e. curvature) is related to the first derivative in the azimuthal coordinates, and the particle’s polar direction is related to the first derivative in the longitudinal coordinates. Since these derivatives have already been computed and sorted in the QC module, we use the average of the two median values (i.e. ignoring the extremum values) of these first derivatives to represent the best track’s curvature and polar direction. These quantities are provided for subsequent trigger decisions.

Similarly, the four crookedness metrics of the best track are also provided by the QC module. Together they serve as a proxy for the $\chi ^2$ of a helical fit to the hit coordinates. These metrics can be used for subsequent rejection of ghost tracks. On the basis of these metrics, studies of the ghost rate have been shown in^3^ to be low enough to meet trigger-bandwidth requirements.

### Event pipeline

**Figure 4:**
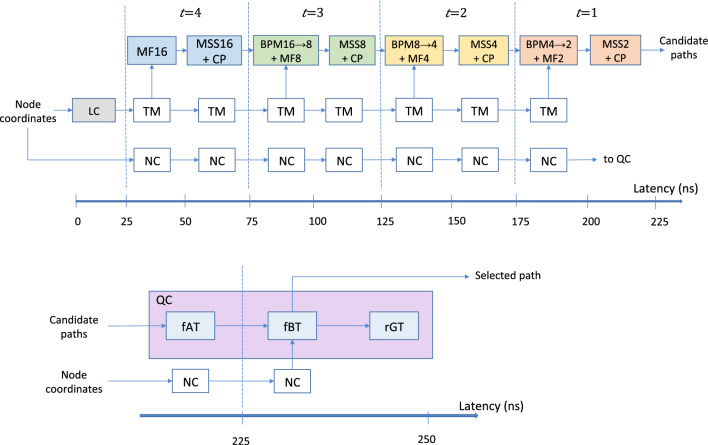
Block diagram of the event-level pipeline. The logic modules are indicated in color and the shift registers for TM and the node coordinates (NC) are indicated by the clear blocks.

The LHC produces new data every 25 ns. To accomplish a real-time processing architecture, we configure the modules into blocks such that each block’s latency is under 25 ns. The pipeline breaks our iterative algorithm into a sequence of smaller tasks to achieve data flow at a rate determined by the slowest task in the workflow. As shown in Fig. 4, the data flow is designed to be unidirectional with no loops or branches and hence amenable to pipelining.

We combine BPM and MF into one block, and MSS and CP into another block, so that together with LC and QC there are four types of blocks constituting the event pipeline. This grouping minimizes the number of pipeline stages, the idle time of the hardware and the total latency of the pipeline, while maintaining the 40 MHz real-time throughput.

When a collision event occurs, data from a wedge of sensors are fed into the LC block. Its output TM is available for the first MF $(t=4)$ before the next event arrives. We implement a “shift register” of TM such that each event’s TM is accessible by all blocks processing that event sequentially (corresponding to $t = 4,3,2,1$). In synchronization, the event’s processed information evolves down the pipeline until the best track is generated $\approx 250$ ns after the raw data were fed into the system. Since there are no loops and branches in this workflow, the event pipeline can process a continuous stream of events indefinitely.

**Figure 5:**
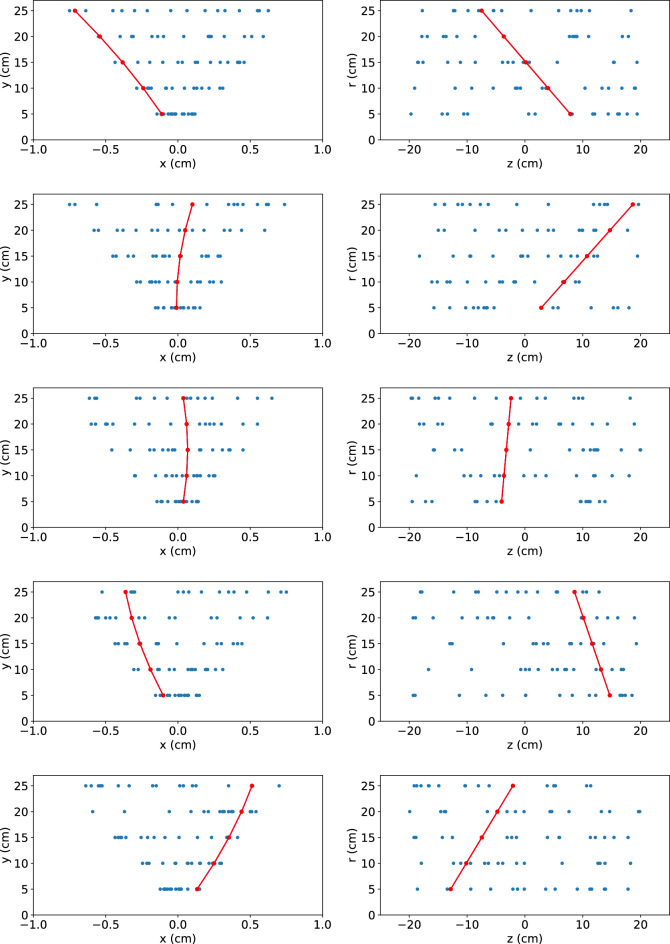
Examples of the track-finding ability of the algorithm, demonstrated on simulated data. The C code used for vitis synthesis is executed as software to emulate the algorithm’s hardware results. The red points represent the hits associated with the high-momentum particle of interest, and the blue points represent hits from random noise. The red curve shows the trajectory identified by the algorithm. The embedded particle has a transverse momentum of 10 GeV/*c* and traverses an axial magnetic field of 2 T.

## Validation

Detailed studies of the physics case for this algorithm and its analytic performance metrics have been presented in^3^. It was shown that, for a 40 MHz beam collision rate with 200 proton-proton interactions per beam collision, the algorithm can achieve a signal efficiency $> 99.9$% and a spurious trigger rate of ${\mathcal {O}}$(10) kHz.

The thrust of this paper is the algorithm’s implementation as a parallelized graph-computing architecture that has a pre-determined latency, throughput and resource usage for a pattern recognition use case that is typically considered to be non-deterministic. Since the algorithm has been re-implemented to deliver on these requirements, we demonstrate the logical consistency of this implementation by executing on simulated data the C code used for vitis synthesis. The data are simulated by embedding the hits associated with a high-momentum charged particle ($p_T > 10$ GeV) within a collection of randomly distributed hits. We implement multiple Coulomb scattering, which deflects the particle direction by an amount dependent on the momentum and the radiation lengths traversed. The latter is 4% for each sensor layer at normal incidence, as in^3^. Assuming 2D pixels of dimensions 50 $\upmu$m $\times$ 50 $\upmu$m, hits are smeared uniformly over a ± 25 $\upmu$m interval in each dimension to emulate digitization. Figure 5 shows examples of the software emulation, illustrating that the circuit logic correctly finds the trajectory of the particle of interest.

**Figure 6:**
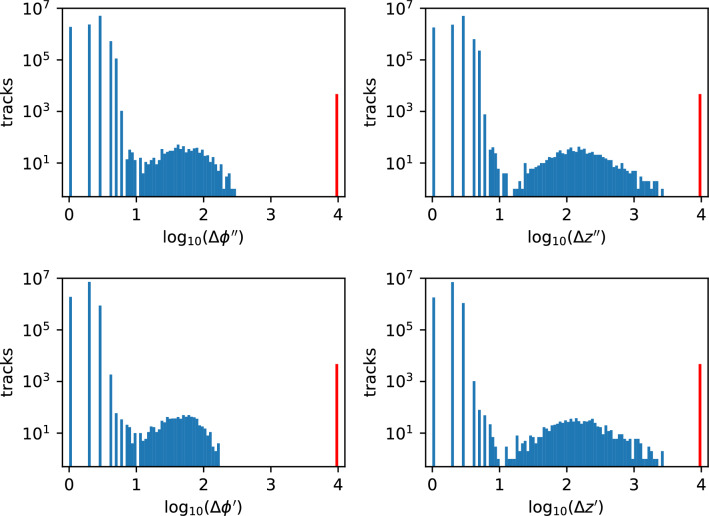
Results of a high-statistics C simulation test. Distributions of the smoothness metrics $\Delta \phi ^{\prime \prime }$ and $\Delta z^{\prime \prime }$ and the consistency metrics $\Delta \phi ^{\prime }$ and $\Delta z^{\prime }$ in the two dimensions respectively are shown for ten million simulated particles ($p_T > 10$ GeV). The distributions are discrete because all hit coordinates and their derivatives are represented as integers. The rate of unreconstructed or poorly reconstructed tracks, which are indicated by a value set to $10^4$ for these metrics (shown in red), is 0.05%.

As mentioned in the sections describing the quality control (QC) module, our circuit returns four quality metrics as well as two physics parameters associated with the best track. The metrics referred to as $\Delta \phi ^{\prime \prime }$ ($\Delta \phi ^{\prime }$) and $\Delta z^{\prime \prime }$ ($\Delta z^{\prime }$) in^3^ quantify the largest difference in the second (first) derivatives along the track. The results of a high-statistics C simulation (Fig. 6) show that the inefficiency of the algorithm on simulated data is 0.05%, and demonstrate the effectiveness of the salient feature of our algorithm; local decisions coupled with information percolation lead to the globally optimal decision.

**Figure 7:**
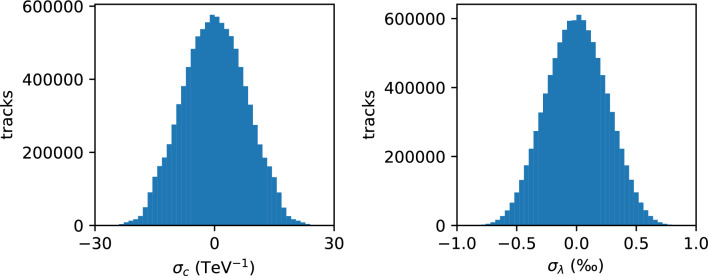
Results of a high-statistics C simulation test. Distributions of the difference $\sigma _c \equiv (c_{\textrm{reconstructed}} - c_{\textrm{truth}})$ and $\sigma _\lambda \equiv (\lambda _{\textrm{reconstructed}} - \lambda _{\textrm{truth}})$ are shown for ten million simulated particles, where *c* refers to the curvature of the trajectory in the azimuthal dimension and $\lambda$ refers to the cotangent of the polar angle in the longitudinal dimension. The curvature is defined as $c \equiv q/p_T$ where *q* is the particle charge and $p_T$ is its momentum component transverse to the beam collision axis. The curvature and $\lambda$ distributions are generated uniformly over the intervals $[-0.1,0.1]$ GeV^-1^ and $[-0.8,0.8]$ respectively. The curvature resolution is 7.9 TeV^-1^ and the $\lambda$ resolution is 0.25 $\textperthousand$.

The fidelity of the algorithm is demonstrated by comparing the curvature and the cotangent of the polar angle of the reconstructed track with the corresponding values for the simulated particle. The comparison (Fig. 7) demonstrates that tracks are reconstructed with the expected resolution and that the rate of non-Gaussian errors is negligible.

**Figure 8:**
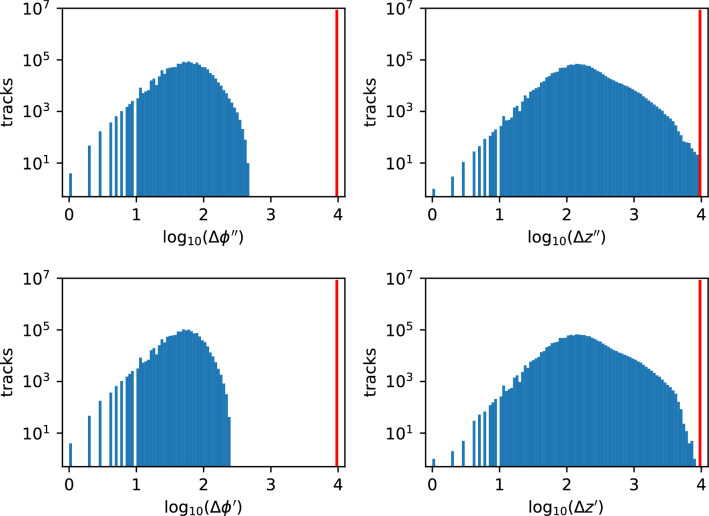
Results of a high-statistics C simulation test on ten million random hit collections, similar to Fig. 6 but without embedding a high-momentum particle of interest. Distributions of the smoothness metrics $\Delta \phi ^{\prime \prime }$ and $\Delta z^{\prime \prime }$ and the consistency metrics $\Delta \phi ^{\prime }$ and $\Delta z^{\prime }$ in the two dimensions respectively are shown. The spurious trigger rate is estimated to be $(0.3 \pm 0.2)$ per million collections, where a trigger track is defined as a reconstructed track with all four quality metrics below the value of 10.

An important aspect of trigger design is the rate of spurious triggers, i.e. reconstructed tracks satisfying the trigger requirements in the absence of a true particle of interest. To estimate the spurious trigger rate for this implementation, we execute the C code on ten million collections of random hits as for Fig. 6, but without embedding a high-$p_T$ particle. The distributions of the quality metrics for (spurious) reconstructed tracks, shown in Fig. 8, are skewed toward large values. We define a trigger track as a reconstructed track whose quality metrics all have values less than 10. This selection requirement is motivated by Fig. 6 where the distributions for correctly-reconstructed particles peak well below the value of 10 ($\log _{10}$[metric]$<1$), but have a second peak well above this value when the algorithm misses one or more correct hits. With this quality requirement, the algorithm’s efficiency is still 99.94% (the inefficiency for true particles increases from 0.05% to 0.06%), and the spurious trigger rate is $(0.3 \pm 0.2_{\textrm{stat}})$ per million wedges. With the $\approx 2000$ wedges needed for coverage of the pixel detector, the expected spurious trigger rate is ${\mathcal {O}}$(0.1%) per bunch crossing or ${\mathcal {O}}$(40 kHz).

Note that the hit resolution assumed above is for single-pixel hits; charge-sharing between adjacent pixels improves the cluster’s position resolution considerably. The performance of our algorithm improves with hit resolution; to illustrate, the study is repeated with a hit resolution improved by a factor of two (*rms* of 7 $\mu$m, as assumed in^3^). For the same quality requirement on the trigger track as above, the inefficiency reduces by a factor of three, to 0.02% and the spurious trigger rate reduces by more than a factor of three, to $< 0.1$ per million wedges or ${\mathcal {O}}$(10 kHz), consistent with the detailed study presented in^3^.

## Discussion

As discussed in^3^, the 2D pixel sensors of the ATLAS and CMS experiments at the LHC would record $\mathcal O$ $(10^5)$ hits every 25 ns. It would require a bandwidth of tens of Tbps to read out this information. An alternate approach is to install the track-finding circuitry on-detector, requiring data transmission over local detector regions only. Off-detector readout would be triggered if a high-momentum track is identified. Our design enables this edge-computing capability; the point cloud would be partitioned into $\mathcal O$ $(1000)$ wedges, each processed by our proposed circuit, all on-detector. Our long-term vision is the implementation of this “smart tracker” with self-triggering capability.

This edge-computing approach will require the slicing algorithm mentioned in the introduction to be implemented as a high-throughput and low-latency circuit which will operate upstream of the track-finder presented here. We note that the LUT and FF usage of the track-finder (shown in Table 1) is 80% and 50% respectively of the resources available on the chosen FPGA. We will investigate the possibility of implementing the slicing algorithm using the remaining resources, to minimize the system’s footprint, power and cooling needs.

The circuit design could be ported from an FPGA to an application-specific integrated circuit (ASIC) to reduce the footprint substantially; however, as FPGAs with higher circuit density become available, a transition to ASICs may be unnecessary. The XCVU19P is fabricated with the integrated-circuit technology node of 16 nm, and 7 nm is expected for the next generation of FPGAs. Radiation tolerance can be achieved by using embedded FPGA (eFPGA) technology to integrate the intellectual property (IP) core of the FPGA into an ASIC.

## Synposis

We summarize the salient features of our track-finding algorithm and its FPGA implementation.

1. Many machine-learning solutions to pattern-recognition problems are based on supervised learning (for examples, see^14^ and references therein), thereby requiring (often large) training samples. Our solution requires no training and can be considered as a form of unsupervised learning.
2. Unsupervised learning methods of pattern recognition or feature extraction may have a data-dependent latency. For example, *k*-means clustering is a popular method of unsupervised learning to partition *p* points into *k* clusters. It has been shown^15^ to have a data-dependent latency that is, in the worst-case, exponential. Our method is explicitly designed to have a fixed latency; it is data-agnostic. This can be a crucial advantage in decision-making applications where time and reliability are both of the essence.

We have demonstrated a partial solution for the challenging use case of real-time track-triggering at the LHC at 40 MHz. Furthermore, the latency is fixed at $\approx 250$ ns which comfortably meets the experiments’ requirement of a few $\mu$s.

## Details of circuit synthesis

We provide a proof of the sort-and-scan engine and a summary of the hardware resource usage.

### Sort and scan engines

We show that the implementation using MF and MSS is equivalent to the original pruning algorithm in^3^. In the original presentation, a list $\mathcal L$ of $n^2$ tuples $(\Box _{ijk,l},j,k)$ at each node (*i*, *l*) is sorted in order of ascending laplacian values $\Box _{ijk,l}$, and the first $\frac{n}{2}$ distinct occurrences of *j* and *k* index values are noted in sets $S_j$ and $S_k$ respectively. For a given node (*i*, *l*), the creation of $S_j$ and $S_k$ is the goal of the pruning algorithm in^3^.

The same sets $S_j$ and $S_k$ can be built from the row and column minima of an $n \times n$ matrix TM, where TM(*j*, *k*) contains the corresponding laplacian value. The smallest laplacian value in row *a* corresponds to the first appearance of $j=a$ in the ascending list $\mathcal L$. Similarly, the smallest TM$(j,k=a)$ in column *a* corresponds to the first appearance of $k=a$ in $\mathcal L$.

Let *M* be the array of the row minima, where *M*(*a*) contains the smallest laplacian value in row *a*. Note that if $M(a) < M(b)$, then the tuple with $j=a$ occurs before the tuple with $j=b$ in $\mathcal L$. Therefore, the *j*-values associated with the smallest $\frac{n}{2}$ row minima are the first $\frac{n}{2}$ distinct *j*-values that appear in $\mathcal L$ i.e. these *j*-values are the elements of $S_j$.

Similarly, the *k*-values associated with the smallest $\frac{n}{2}$ column minima are the first $\frac{n}{2}$ distinct *k*-values in $\mathcal L$, i.e. these *k*-values are the elements of $S_k$. The logic of the previous paragraph is symmetric between rows and columns, since it can equally well be applied to the transpose of TM.

### Resource usage

We summarize the resource usage by the LC, MF, MSS and QC modules as estimated by vitis hls. Table 1 shows the timing characteristics and resource usage of the various blocks, modules and their constituent pipelined functions.

Per Table 1, the LC module contains three pipelined functions, (1) LC_$(D+D)$ adds two doublets *D*, where each doublet of integer coordinates represent a 2D spacepoint, (2) LC_$(16D+D)$ adds a doublet to an array of 16 doublets in parallel, and (3) LC_(16U$+$) performs pairwise addition on two arrays of 16 integers, in parallel, after computing their respective absolute (unsigned) values, also in parallel. The last function in the LC module, LC_ET, encodes each laplacian value and the corresponding node indices into a 24-bit word. Other labels used in Table 1 are described in the table caption.

The choice of $L=5$ pixel layers (planned for the ATLAS and CMS experiments at the HL-LHC) may be replaced by silicon strip detectors at larger radii or planar geometries at fixed-target experiments. The relevant parameter $(L-2)$ represents the number of intermediate layers at which graph computing is performed (excluding the first and last layer). Our circuit may be deployed on a subset of the layers, upon considerations of occupancy and acceptance. Table 2 shows the resource usage according to vitis synthesis for different numbers of intermediate layers. Excluding the quality-control module, the usage for the rest of the circuit is proportional to $(L-2)$, as expected since the other modules are repeated for each intermediate layer.

**Table 2:** Resource usage according to vitis hls 2020.2 synthesis for three values of $(L-2)$, the number of intermediate sensor layers. The quality-control module is excluded from these syntheses because its resource usage scales differently with $(L-2)$. The usage for the rest of the circuit is proportional to $(L-2)$, as expected since the other modules are repeated for each intermediate layer.

Resource type	$L-2$		
	1	2	3
Digital signal processors	1,312	2,624	3,936
Flip-flops	1,336,457	2,623,670	3,929,414
Lookup tables	912,402	1,931,541	2,832,108

#### Minimum Finder

**Figure 9:**
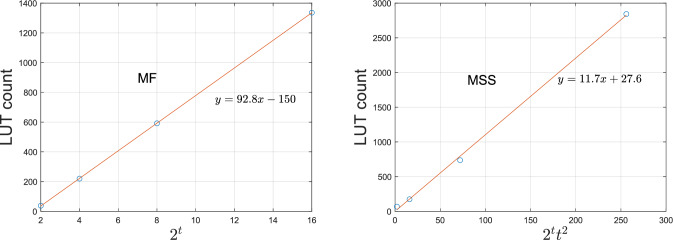
(left) LUT usage of the synthesized MF module as a function of $2^t$, the number of inputs. (right) LUT usage of the synthesized MSS module as a function of $2^t t^2$, where $2^t$ is the number of inputs to be sorted. The open circles show the estimates from vitis hls for $t \in \{1, 2, 3, 4\}$ respectively. The line represents the best linear fit to the point estimates.

Finding the smallest of $2^t$ numbers requires *O*(*t*) sequential stages and clock cycles. The circuit implementation needs $O(2^t)$ comparators. The number of lookup tables used by vitis hls for the MF module is shown in Fig. 9 for $t \in \{1, 2, 3, 4\}$.

#### Minimum Set Selector

Sorting $2^t$ numbers requires $O(t^2)$ sequential stages and clock cycles. The circuit implementation needs $O(2^t t^2)$ comparators. The number of lookup tables used by vitis hls for the MSS module is shown in Fig. 9 for $t \in \{1, 2, 3, 4\}$.

## Data Availability

The dataset used and analysed during the current study is available from the corresponding author on reasonable request.
